# Aortic valve visualization and pressurization device: a novel device for intraoperative evaluation of aortic valve repair procedures

**DOI:** 10.1093/ejcts/ezad291

**Published:** 2023-08-23

**Authors:** Bardia Arabkhani, Stefan C Sandker, Jerry Braun, Jesper Hjortnaes, Thomas J van Brakel, Dave R Koolbergen, Robert J M Klautz, Mark G Hazekamp

**Affiliations:** Department of Cardiothoracic surgery, Leiden University Medical Center (LUMC), Leiden, Netherlands; Department of Cardiothoracic surgery, Leiden University Medical Center (LUMC), Leiden, Netherlands; Department of Cardiothoracic surgery, Leiden University Medical Center (LUMC), Leiden, Netherlands; Department of Cardiothoracic surgery, Leiden University Medical Center (LUMC), Leiden, Netherlands; Department of Cardiothoracic surgery, Leiden University Medical Center (LUMC), Leiden, Netherlands; Department of Cardiothoracic surgery, Amsterdam UMC, Amsterdam, Netherlands; Department of Cardiothoracic surgery, Leiden University Medical Center (LUMC), Leiden, Netherlands; Department of Cardiothoracic surgery, Amsterdam UMC, Amsterdam, Netherlands; Department of Cardiothoracic surgery, Leiden University Medical Center (LUMC), Leiden, Netherlands

**Keywords:** Valve-sparing root replacement, Aortic valve repair, Intraoperative, Device

## Abstract

**OBJECTIVES:**

Aortic valve repair procedures are technically challenging, and current intraoperative evaluation methods often fail to predict the final echocardiographic result. We have developed a novel intraoperative aortic valve visualization and pressurization (AVP) device, enabling valve inspection under physiological conditions, and measuring aortic valve insufficiency (AI) during cardioplegic arrest.

**METHODS:**

The AVP device is attached to the (neo)aorta, after any type of aortic valve repair, while the heart is arrested. The root is pressurized (60–80 mmHg) using a saline solution and an endoscope is introduced. The valve is inspected, and the amount of valvular leakage is measured. Postoperative ‘gold standard’ transesophageal echocardiogram measurements of AI are performed and compared against regurgitation volume measured.

**RESULTS:**

In 24 patients undergoing valve-sparing root replacement, the AVP device was used. In 22 patients, postoperative echocardiographic AI was ≤ grade 1. The median leakage was 90 ml/min, IQR 60–120 ml/min. In 3 patients, additional adjustments after visual inspection was performed. In 2 patients, with complex anatomy, the valve was replaced. In one, after evaluation with the device, there was undesirable result visually and residual AI of 330 ml/min, and in another, 260 ml/min residual AI was measured and valve restriction on visual inspection.

**CONCLUSIONS:**

The novel AVP device enables intraoperative evaluation of the valve under physiological conditions, while still on arrested heart, and allows for targeted adjustments. The AVP device can be an important aid for intraoperative evaluation of the aortic valve, during valve repair and valve-sparing procedures, thereby making the operative result more predictable and the operation more efficient.

**Figure:**
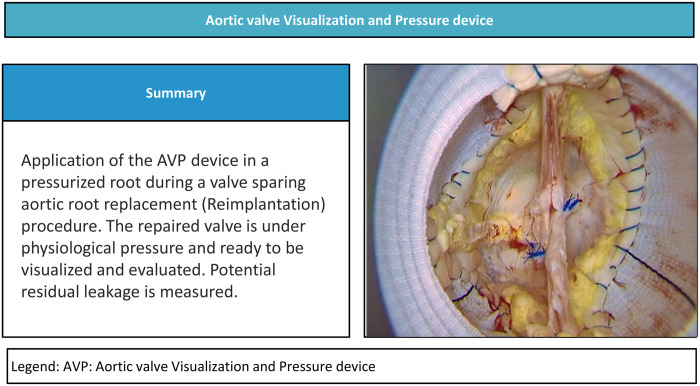

## INTRODUCTION

Valve-sparing aortic root replacement (VSRR) procedures are the preferred treatment in patients with ascending aorta aneurysm and/or aortic valve regurgitation, especially in younger patients, according to the EACTS/ESC guidelines [1]. Although there is accumulating evidence that valve-sparing procedures are associated with better outcome compared to composite valve replacement procedures [2], still the majority of ‘repairable’ valves are replaced [3]. This may be due to the complexity of valve-preserving procedures, which are more time consuming (i.e. longer bypass and aortic cross-clamp times) than valve replacement procedures. In addition, the outcomes are sometimes unpredictable and compared to mitral valve repair, the equivalent of a pressurized ‘water test’ is lacking. Nevertheless, valve-sparing procedures are gaining popularity and a growing number of centres currently have dedicated ‘aortic root surgeons’ interested in these procedures.

To date, evaluation of the repair is done by a transoesophageal echocardiogram (TOE), after declamping the aorta and weaning from cardiopulmonary bypass. In case of unsatisfactory results, another period of cardioplegic arrest is needed to readdress the valve or replace it. Hence, there is an unmet need for an intraoperative tool to evaluate the valve under physiological conditions after repair. Preferably this should be done in a pressurized aortic root to mimic the physiological situation on the arrested heart. Several methods have been proposed for intraoperative evaluation [4, 5], however all in porcine (simulation) models, and there has been no routine clinical use so far.

We have developed a novel intraoperative Aortic valve Visualization and Pressurization (AVP) device (patent ref. Nr. P307644), which allows for intraoperative evaluation of the aortic valve during cardioplegic arrest in valve-sparing procedures, while the aortic root is under ‘physiological’ pressure. The evaluation takes place before reimplantation of the coronary arteries into the Dacron graft or native root. Additionally, the AVP device allows for the exact measurement of any residual transvalvular insufficiency. The visual aspect under physiological conditions together with information on valvular leakage volume provides the information needed by the surgeon to predict and evaluate the function of the repaired valve.

## MATERIALS AND METHODS

### Ethics statement

This study was approved by The Institutional Review Board of the Leiden University Medical Center. Informed consent was waived (study ID: P16.004).

### *The* aortic valve visualization and pressurization *device*

The AVP device is a conical tube with 1 end cap connecting to the native aortic root or a vascular aortic prosthesis. The other end contains 2 connection ports. One port for connection of a line, which is usually the connector tube that is used for cardioplegia, is connected to the heart-lung machine. The second port allows for the introduction of an endoscopic camera. Any commercially available scope with an outer diameter of approximately 10 mm will be applicable. We advise to use a 30-degree angle camera to be able to evaluate the valve from an angle to better assess the cusps, but a 0-degree camera may also be used. The device may be used in any type of VSRR procedures (reimplantation and remodelling), in supracoronary ascending replacement or (wrapped) Ross procedures, and even in aortic valve repair without using a vascular graft.

**Figure 1:**
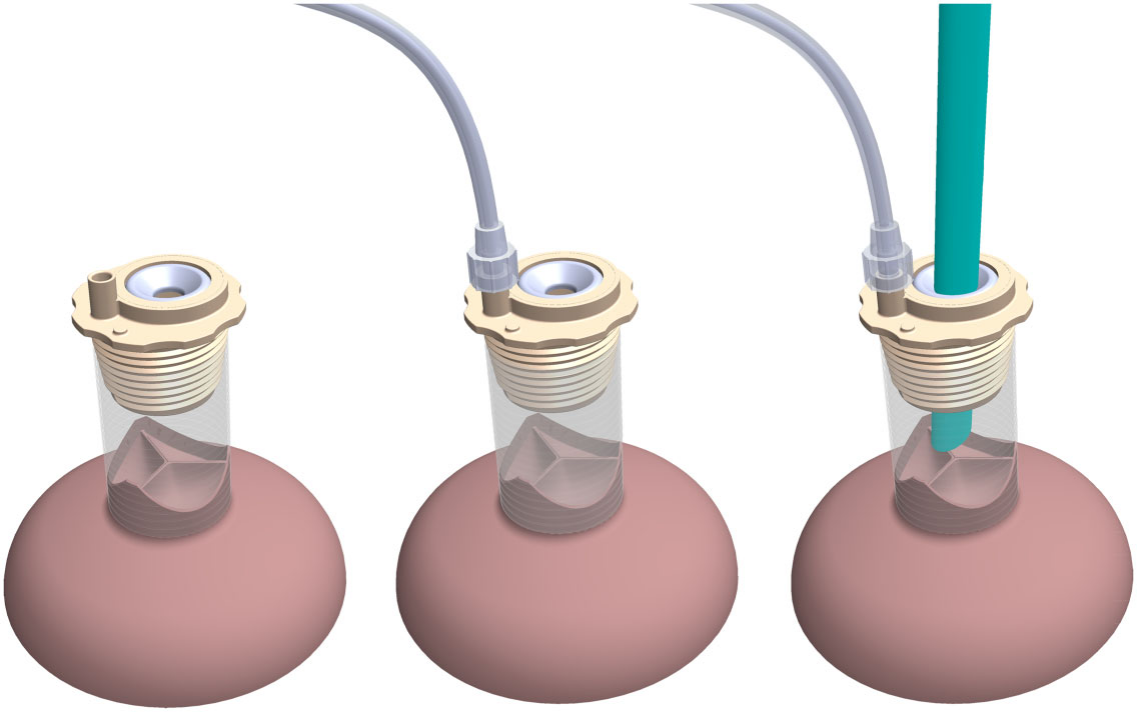
Schematic application of the aortic valve visualization and pressurization device during a valve-sparing root replacement procedure. Left: attachment of the device to a tubular graft. Mid: attachment of a line to pressurize the root with saline solution. Right: introduction of a video-endoscope.

**Figure 2:**
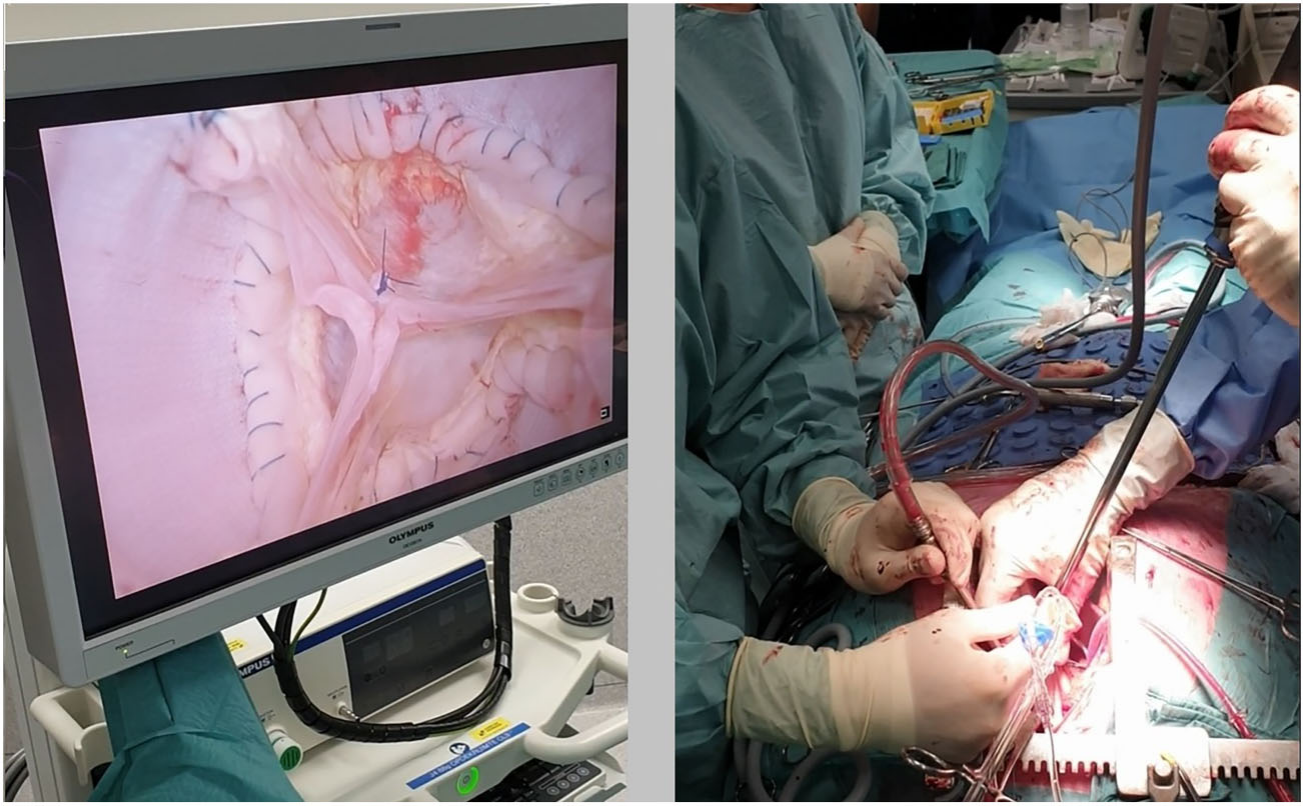
Picture of visualization of the aortic valve by the aortic valve visualization and pressurization (AVP) device during a valve-sparing aortic root replacement (reimplantation) procedure in a patient.

In VSRR procedures, the first step is to connect the AVP device to the tubular (Dacron) graft, directly after reimplantation of the valve into the Dacron graft (in reimplantation technique) or attachment of the Dacron (in remodelling technique), before reimplantation of the coronary arteries. The next step is to connect the cardioplegia line to the appropriate port of the device. A physiological saline solution (0.9% NaCl) is then applied to ‘wash’ the erythrocytes from the valve and to de-air the aortic neo-root. Hereafter, the endoscopic camera is introduced, and the root is pressurized by using saline solution of up to 70 mmHg, comparable to administration of cardioplegia. The next step is to evaluate the aortic valve under diastolic pressure. Any potential prolapse or retraction of the valve may be observed and if necessary, any adjustment is possible after removing the device. The last step is to reconnect the device and measure the amount of valvular leakage for 1 min, by pressurizing the root, while maintaining a constant flow. Figure 1 shows a schematic application of the AVP device in a VSRR procedure. In aortic valve repair procedures, the AVP device can be attached to the native aorta just above the STJ. In case of a perfectly sufficient valve, there should be no (valvular) leakage, except for the leakage due to the porosity of the tubular graft. This leakage, in an experimental set-up for prosthesis size 26 and 30 mm, was estimated to be approximately 1.72 ± 0.02 and 3.34 ± 0.03 ml/cm^2^/min (depending on graft surface area), respectively. The leakage at the site of the AVP device connection reached a maximum of 4.9 ± 0.58 ml/min. Figure 2 shows a picture of visualization of the aortic valve by the AVP device during a VSRR procedure in a patient. The process of applying the AVP device in a VSRR (reimplantation) procedure is displayed in Video 1.

## RESULTS

### Clinical results and valvular leakage measurements

Since 2019, in a total of 24 patients undergoing a valve-sparing root replacement (reimplantation) procedure, the AVP device was used to evaluate the aortic valve after repair. After the evaluation and eventual adjustments of the valve, the coronaries were implanted in the prosthesis and the prosthesis was anastomosed to the distal ascending aorta. After declamping and weaning from CPB, the valve was evaluated by means of a structural TOE, performed by a cardiologist. The postoperative residual aortic valve insufficiency (AI) grade was used for the correlation with the amount of leakage measured by the AVP device. The median leakage measured by means of the AVP device was 90 ml per minute (IQR 60–120 ml/min; mean 77 ml/min; range 25–180 ml/min).), with the root pressurized to 60–70 mmHg. In 22 patients, the intraoperative TOE measurements showed an AI grade of 1 or less. Notably, in 2 cases, the aortic valve was replaced after the first visual inspection by the AVP device, due to structural anomalies of the valve, deemed not repairable. The first patient, with an asymmetric bicuspid valve on visual inspection using the AVP device, there was a lower coaptation height of the ‘fused’ cusp and a valvular leakage of 260 ml/min was measured. Echocardiography showed a grade 2 AI, and the valve was deemed not repairable and was replaced. The second patient also had a more complex unicuspid valve anatomy. On visual inspection, using the AVP device, there was retraction with very little cuspal tissue. We measured a leakage of 330 ml/min. After 1 attempt to adjust and repair the valve by central plication, the valve was replaced by a biological root prosthesis, due to unsatisfactory result of the repair (significant residual AI).

**Figure 3:**
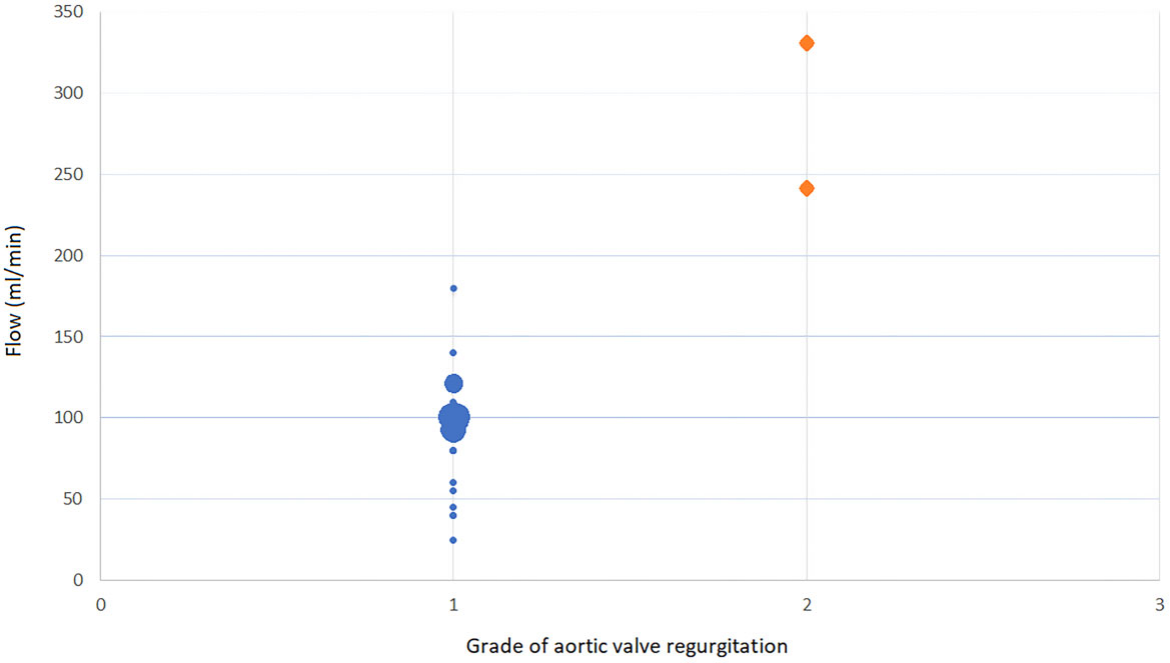
Intraoperative measurements of aortic valve regurgitation in ml/min and postoperative transoesophageal echocardiogram measured grade of aortic valve insufficiency.

In 3 patients, additional adjustments were performed after visual inspection of the valve through the AVP device, with successful results. In 2 patients, there was a leakage of 180 ml/min measured, and in another 140 ml/min. After adjustment, this was reduced to 60 and 90 ml/min. In 1 patient the visual inspection showed an uneven closure line (leakage 170 ml/min), and the result was predicted to be uncertain, but the surgeon decided to declamp and evaluate by TOE without additional adjustments. The TOE showed unacceptable AI and adjustments by means of central plications were made during a second cross-clamp time. Now, the AVP device showed a much better visual result, and the post-pump echo showed a functionally perfect valve. Figure 3 shows the intraoperative transvalvular AI flow measurements, directly after repair, through the AVP device, and the postoperative TOE measurements. On average, it took 3–4 min to attach the device and visualize, and to do the measurements.

## DISCUSSION

Evaluation of aortic valve function in valve-sparing aortic root replacement and valve repair procedures in non-physiological state is difficult while intraoperative inspection of the repaired valve under physiological conditions will improve operative results. The AVP device can provide lacking information on valve coaptation and geometry while still on cardioplegic arrest. This may help to predict the result of the repair, and by making the operation more efficient it may lower the threshold to perform valve-sparing procedures. The AVP device will usually be used in valve-sparing root replacement procedures, although it could also be useful in other procedures such as the Ross operation, isolated aortic valve repair and supracoronary ascending aortic replacement.

Since the 1990s, when valve-sparing procedures were introduced, there have been various tools introduced in experienced centres to guide less experienced surgeons towards a structural approach in VSRR procedures [6–8]. Additionally, several approaches to mimic the diastolic state of the aortic valve after repair are proposed such as pressurizing the new aortic root by administering cardioplegia through the distal end of the graft with partial clamping and the ‘El Khoury’s kiss’ [9]. Nevertheless, these techniques do not allow visual inspection under physiological pressure. Pressurization of the neoaortic root and visualizing the valve during valve-sparing procedures while still on cardioplegic arrest, will allow the surgeon to perform targeted adjustments on the valve, when necessary, with the aim to reduce the need for re-clamping. Moreover, since the coronary arteries are still not attached to the graft, it is less time-consuming to perform a composite root replacement (Bentall) procedure in case of unsatisfactory results, without the need to close and declamp the aorta, wean the patient off CPB and wait for the echocardiographic assessment.

In addition, measuring the exact amount of valvular regurgitation will provide additional information on the success of the repair. However, more data are needed to accurately correlate residual AI (ml/min) measured by the AVP device, to the intraoperative echocardiographic measurement of residual AI (grade). In other words, we need to validate these AVP measurements, after which we can predict the grade of residual AI visualized by echocardiography, while at the same time making echocardiographic measurements more accurate by assigning volumes to gradations of valve insufficiency.

### Challenges and future perspective

Besides visual inspection and measurements of any residual AI, the echocardiographic evaluation of the aortic valve will still be required as for now there is no precise validation of the amount of regurgitation per time unit to ‘gold standard’ echo measurements. Ideally, there should be measurements of several grades of aortic valve regurgitation, and this should correlate perfectly with the amount of regurgitation measured by means of the AVP device. However, intraoperative echo measurements on the arrested heart are very difficult. We have tried this in a few cases, by filling the left ventricle and using colour Doppler, but it remains complex to evaluate the valve. Moreover, the porosity of the Dacron (usually Terumo’s Valsalva or Getinge’s Cardioroot) graft makes the interpretation of the amount of leakage through the valve and its correlation to grade of regurgitation on echo less accurate. Nevertheless, the amount of leakage per cm^2^ graft surface area is provided by the manufacturers, and we found comparable results during assessments in our lab. By measuring the length of the graft and the diameter, one should be able to predict the amount of leakage per minute due to graft porosity. This will help to interpret the measurements resulting from using the AVP device more accurate.

Another critical issue is the ease and speed of application of an intraoperative device. If such a device is too hard to apply, this could lead to unwanted additional CPB- and aortic cross-clamp times. Moreover, if the sealing of the device to the graft is not ‘watertight’, transvalvular AI volume measurements may be less accurate, leading to misinterpretation of the results. However, the AVP device allows for perfect sealing of the graft, hence accurate measurements of potential residual AI. The device is also quite easy and fast to attach to and detach from different graft sizes, and taking less than a minute.

In conclusion, the AVP device provides a safe and straightforward way to intraoperatively visualize and evaluate the repaired aortic valve on the arrested heart, under physiological conditions, and allows for measurements of any transvalvular leakage. This will allow targeted adjustments when necessary and will guide surgeons towards a more reliable and structural evaluation of the valve repair.

## Data Availability

Data will be shared on request to the corresponding author.

## ABBREVIATIONS

AI: Aortic valve insufficiency
AVP: Aortic valve visualization and pressurization
CPB: Cardiopulmonary bypass
TOE: Transoesophageal echocardiogram
VSRR: Valve-sparing aortic root replacement
